# Australian compilation of seismic-derived bathymetry

**DOI:** 10.1038/s41597-025-04512-z

**Published:** 2025-02-06

**Authors:** Ulysse Lebrec, Victorien Paumard, Juliette Denudt, Catherine Du Réau, Simon C. Lang, Julien Bailleul

**Affiliations:** 1 Centre for Energy and Climate Geoscience, School of Earth and Oceans, The University of Western Australia, 35 Stirling Highway, Perth, WA 6009 Australia; 2 U2R 7511, Basins-Reservoirs-Resources (B2R), Geosciences Department, UniLaSalle – University of Picardie Jules Verne, 19 rue Pierre Waguet, Beauvais, 60000 France

**Keywords:** Ocean sciences, Geophysics

## Abstract

This article presents a national seismic-derived bathymetric compilation based on the integration of 253 3D seismic surveys. Individual surveys were combined to produce four regional compilations covering an area of 267,000 km^2^, with a spatial resolution of 30 × 30 m and a vertical accuracy of 5 m + 5%d. The production of the dataset is based on the integration of seismic survey first returns with seismic vessel echosounder measurements. Following the extraction of the depth soundings, all data points were converted from time to depth using synthetic velocity profiles and filtered to remove erroneous records. The seismic survey’s first returns were corrected using navigation depth soundings to account for geometric distortions. All depth values were reduced to WGS84 and EGM2008 datum. A comparison of the seismic-derived bathymetry with multibeam echosounder surveys suggests that where a thin layer of loose sediments overlies a lithified substratum, the seismic first return captures the top of the substratum.

## Background & Summary

High-resolution bathymetric datasets are critical for the exploration of the marine environment^1^. They can be used to map the seafloor and better understand marine habitats but are also a key input for the assessment of ground conditions for offshore infrastructure^2–4^. The latter has become increasingly important in recent years with the exponential development of marine renewable energy with leases covering thousands of square kilometres^5,6^. In this context, the GEBCO Seabed 2030 project was announced in 2021 as part of the United Nations Decade of Ocean Science for Sustainable Development with the aim to map the world’s oceans by the end of the decade^7^. Significant efforts are needed to achieve such an ambitious objective given that, as of 2017, 82% of the 30 arc second GEBCO grid cells did not contain depth measurements^8,9^ and only six percent of the oceans had been surveyed using high-resolution techniques^7^.

Australia, thanks to contributions from the wider AusSeabed community, is leading the effort with approximately 25% of its territorial waters covered by publicly available high-resolution bathymetry^3,10^. However, given the current capabilities, several decades would be required to achieve full coverage, especially considering the growing demand for survey vessels from commercial projects. In this context, several authors have produced regional high-resolution bathymetric compilations by integrating multibeam echosounder and LiDAR surveys with datasets from multiple sources that were not initially acquired to produce bathymetric grids such as satellite imagery, 3D seismic surveys, hydrographic depth soundings, and crowd-sourced singlebeam echosounders^2,11–14^.

**Fig. 1:**
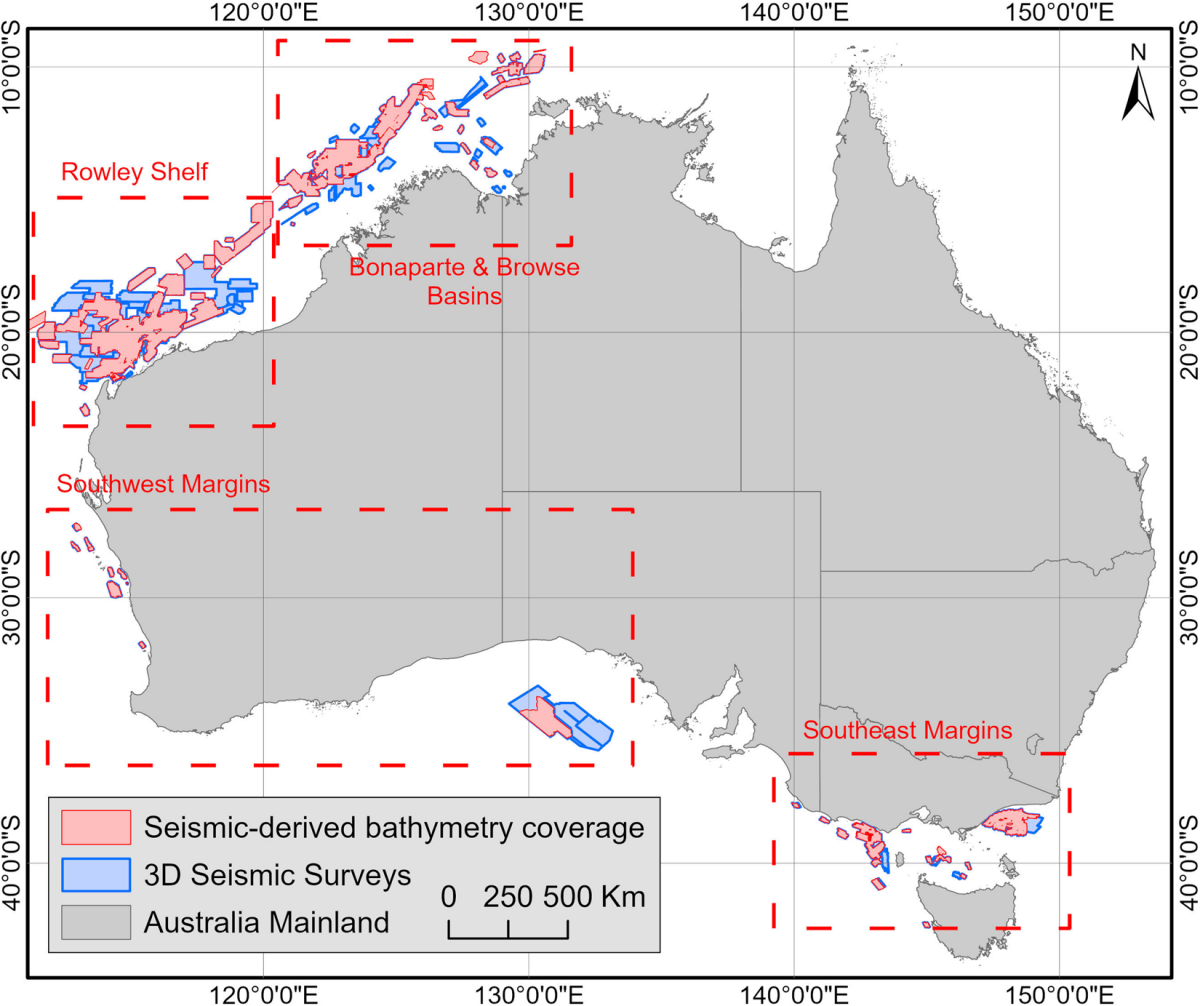
Extent of Australian 3D Seismic surveys that are listed on NOPIMS (blue) and were available for this project (red). Unavailable surveys are primarily confidential.

Seismic surveys, in particular, provide a unique source of accurate and closely spaced depth soundings over large areas^15,16^ that are not dependent on water turbidity or limited by water depth. Over the last forty years, more than 350 3D seismic surveys have been acquired in Australia, covering a combined area of almost 450,000 square kilometres (Fig. 1). Except for multi-client proprietary surveys, most of these 3D seismic data are available from Geoscience Australia’s National Offshore Petroleum Information Management System (NOPIMS). They are commonly used for subsurface interpretation with applications for the industry (e.g., petroleum system element location and characterisation for resource exploration or carbon sequestration) and academia (e.g., reconstruction of palaeoenvironments, climate and sea level^17^). More recently, these surveys have been used for studies of seafloor geomorphology^18–22^ but have so far been scarcely used to produce stand-alone bathymetric datasets. In fact, only 26 seismic reflection surveys have been included in Australian bathymetry compilations^2^, in part due to the software and data storage requirements necessary to process and calibrate 3D seismic data.

In this study, the complete catalogue of publicly available Australian 3D seismic surveys has been reprocessed to produce a national seismic-derived bathymetric data asset with a resolution of 0.0003 degrees (30 m at the equator). The purpose of this manuscript is to present the dataset and the processing steps required to produce this output, including the associated vertical and horizontal accuracies and uncertainties. The dataset is publicly available on the AusSeabed data portal and includes regional compilations over four main areas of interest.

## Methods

### Overview

**Fig. 2:**
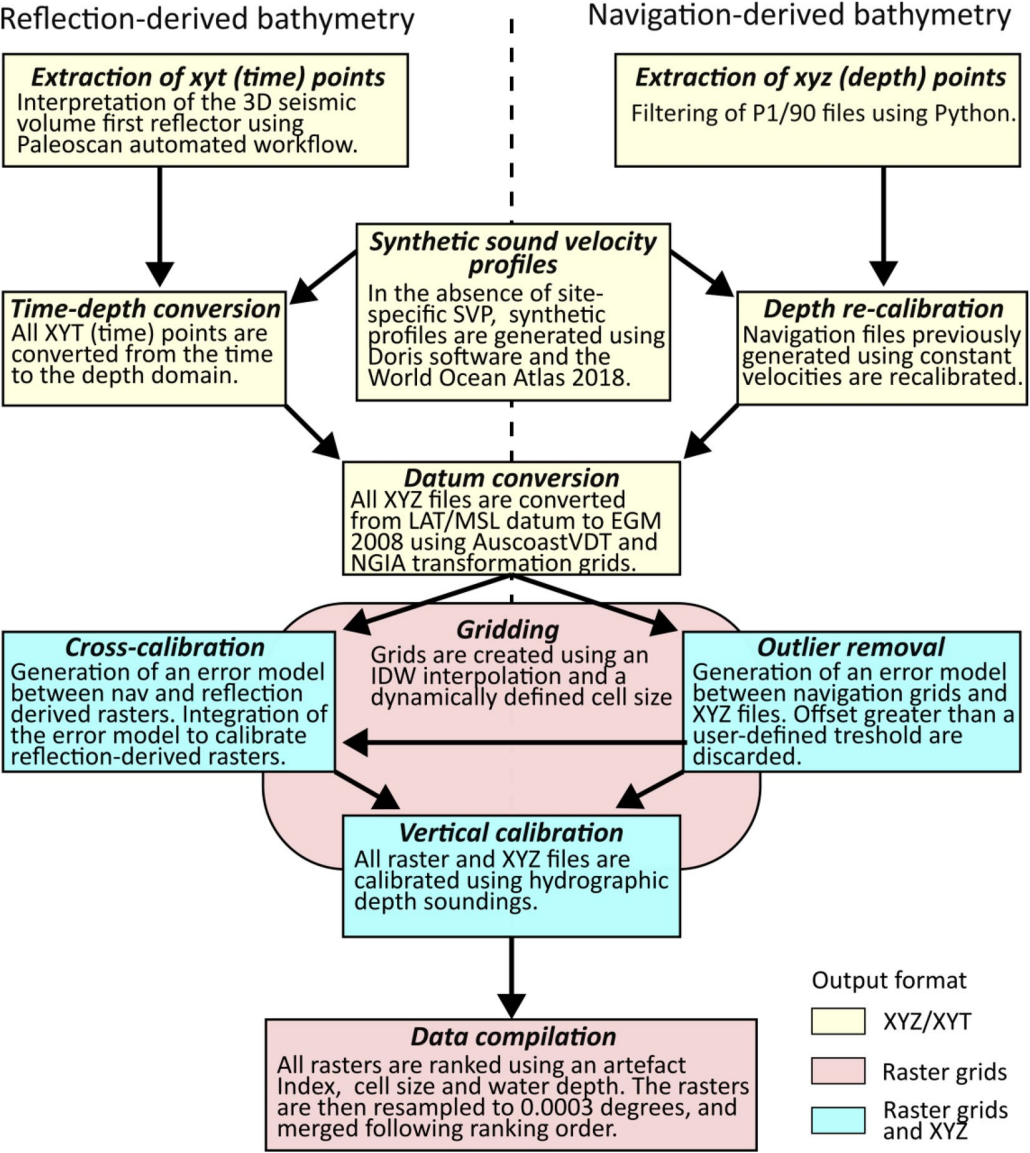
Method overview.

The generation of seismic-derived bathymetry is based on the integration of the first return of reflection data with the associated navigation depth soundings, which are referred to as reflection-derived bathymetry and navigation-derived bathymetry, respectively. The processing is carried out in four main steps including: (1) the extraction of the depth points; (2) the conversion of depth points from the time domain to the depth domain; (3) the preparation of raster grids; and (4) the calibration of the bathymetry (Fig. 2).

### Source data

The source datasets consist of 253 unique 3D seismic surveys and include 233 full-stack time migration surveys in sgy format and 226 navigation records in P1/90 format, as defined by the The Surveying and Positioning Committee^23^. The discrepancy between the number of surveys, sgy files and P1/90 files illustrates the presence of either incomplete or corrupted files that could not be sufficiently recovered to provide meaningful results.

### Extraction of depth points

#### Navigation-derived XYZ points

Navigation data are recorded during the acquisition of 3D seismic surveys and are stored in accordance with the P1/90 standard. The data include, for each shot point, the coordinates of the vessel, seismic sources, seismic receivers and vessel echosounders, each associated with the echosounder depth (metres) measurements^23^. The navigation files were filtered using Python scripts to retain only the echosounder coordinates and depth soundings. Where the echosounder coordinates were not specified, the position of the vessel was used instead. Such filtering is essential because all seismic acquisition elements whose coordinates are listed in the navigation files are assigned, for a given shot point, to the water depth measured at the echosounder location. This can lead to inaccurate depth-sounding – coordinate pairs and, in turn, banded patterns due to XY coordinate offsets^2^.

**Fig. 3:**
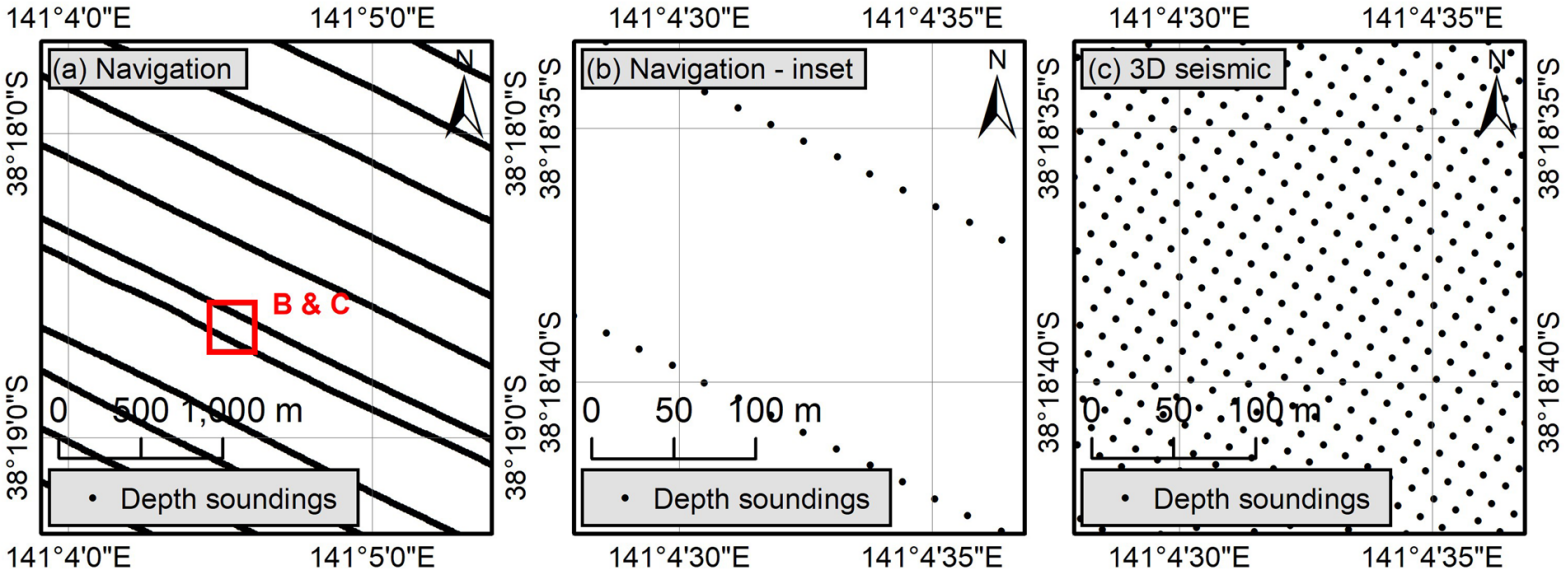
Distribution of depth soundings from P1/90 vessel navigation recordings (**a,b**) and from 3D seismic reflection files (**c**). Note the anisotropy in the distribution of navigation points.

Since 3D seismic surveys rely on multiple streamers^24^ while navigation measurements are tied to the position of the vessel, navigation depth soundings exhibit strong spatial anisotropy: they are recorded every few metres along acquisition corridors (sail lines) that are separated by several times this distance (Fig. 3a,b). The degree of anisotropy has increased over the years, in line with the multiplication of the number of streamers. Indeed, seismic surveys acquired in the 90 s relied on one streamer and therefore on closely spaced sail lines whereas modern surveys can have in excess of 16 streamers^25,26^ resulting in gaps of up to 1600 m between sail lines, given a streamer spacing of 100 m^24^.

#### Reflection-derived XYT

During seismic acquisition, the first return of reflected sound waves shows the first increase in acoustic impedance below the water column which is interpreted as the seabed^27^. This surface, often referred to as the First Pick or First Return, was extracted, in two-way time, using PaleoScan™, a semi-automated full-volume seismic interpretation software following the method of Paumard, *et al*.^28^. The workflow uses similarities between adjacent seismic traces to identify changes in seismic impedance and, in turn, seismic horizons such as the seabed. The resulting measurements are extracted from seismic traces and are therefore not tied to the position of the vessel. They have a typical point spacing that ranges from 12.5 to 25 m (Fig. 3c).

### Time-depth conversion

Seismic reflection datasets are available in the time domain and need to be converted to the depth domain. Similarly, navigation depth soundings, although reported in metres, were often processed using a constant nominal sound velocity of 1,500 m/s and therefore needed to be recalibrated. Seismic velocity stacks which are used to convert seismic traces from the time domain to the depth domain are typically based on a single sound velocity within the water column and are therefore inadequate for bathymetric applications. In addition, CTD measurements are rarely available for legacy surveys. As a result, in the absence of site-specific sound velocity profiles, the conversion of the measurements relied on synthetic sound velocity profiles generated using the Doris software and the World Ocean Atlas 2018 database, which provides monthly predictions of both salinity and temperature values across 102 depth layers with a resolution of 0.25 arc degrees^29,30^.

**Fig. 4:**
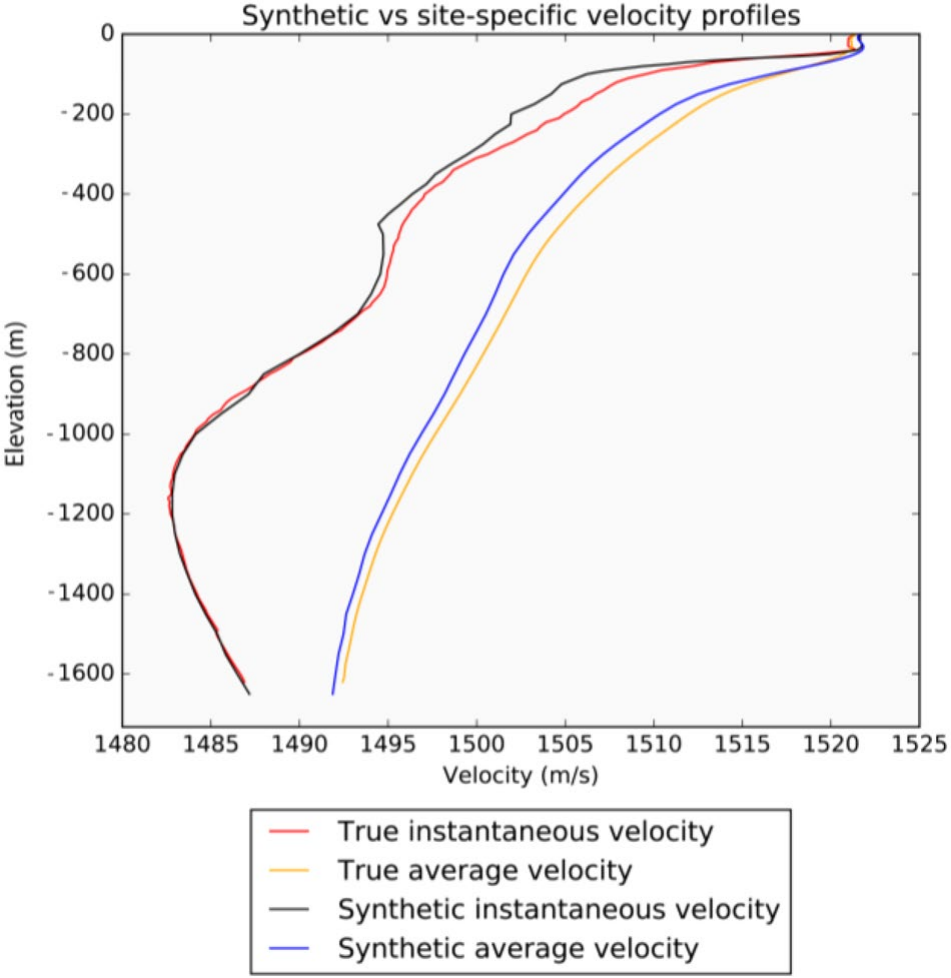
Comparison of synthetic and site-specific velocity profiles at Trim 3D location.

To ensure the best accuracy, a synthetic sound velocity profile was generated for each individual seismic survey, using the coordinates of the deepest point and its acquisition date (Fig. 4). In some cases, the synthetic velocity profiles do not cover the full depth range of the file being converted (e.g., a profile extending to a depth of 100 m for a survey containing navigation depth soundings deeper than 150 m). This discrepancy is related to the variable accuracy of the bathymetry (GEBCO) used in the World Ocean Atlas model to constrain depth layers. In such instances, where the difference in depth was greater than 10%, a regional synthetic sound velocity profile was used instead. Given that these profiles represent instantaneous velocities versus depth, they were first converted to average velocities versus depth and then average velocities versus two-way time before being applied to the time-domain depth measurements using the polynomial equation of the resulting profiles. The degree of the polynomial equations was automatically defined within a range of 1 to 50 to obtain the highest coefficient of correlation between the equation and the underlying data points.

The accuracy of the method was evaluated by comparing depth values obtained using the Trim 3D survey site-specific sound velocity profile and its synthetic counterpart (Fig. 4). On average, over a range of 2 seconds two-way time (twt) with an interval of 10 ms, the depths obtained using the synthetic and true sound velocity profiles show an absolute difference of 0.06%, hence confirming the robustness of the approach. These results are particularly striking in water depths of less than 100 m, where the synthetic profile is associated with an average error of 0.022% whereas a constant velocity of 1,500 m/s results in an average error of 1.31% compared to true sound velocity profile.

### Datum

The seismic surveys used in this study were acquired over a period of 40 years and are based on a variety of vertical and horizontal datums. To ensure a seamless integration, all datasets were reduced to WGS84 horizontal datum and EGM 2008 vertical datum (*sensu* Pavlis, *et al*.^31^), which is now recommended by Geoscience Australia in place of tidal datums. Spatial projections were performed using ArcGIS built-in transformations while vertical adjustments required a combination of methods due to the lack of readily available transformation grids. Firstly, the AusCoastVDT software was used to generate transformation grids from either Mean Sea Level (MSL) or Lowest Astronomical Tide (LAT) to the Ellipsoid (GRS80) which were subsequently combined with the National Geospatial-Intelligence Agency ‘s Ellipsoid to EGM2008 transformation grid^32^ sourced through Geoscience Australia. The resulting LAT to EGM2008 and MSL to EGM2008 transformation grids were then used to transform the data.

**Fig. 5:**
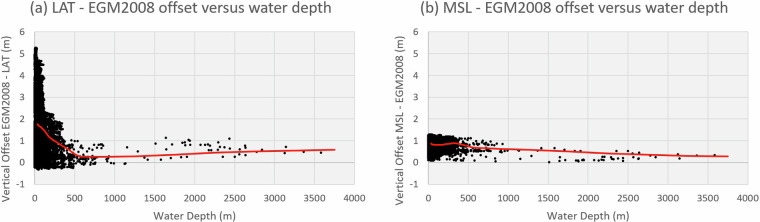
Datum comparison. The red line corresponds to a moving average with a period of 5.

The AusCoast VDT grids extend to approximately the 500 m isobath^33^, limiting the number of surveys that could be converted in this way. However, a comparison of the difference between EGM 2008, LAT and MSL vertical datums as a function of water depth using nearly 200,000 Australian Hydrographic Office (AHO) depth soundings (Fig. 5) shows that their respective differences decrease drastically beyond a depth of 400 m and that vertical offsets become almost constant, likely due to the reduced influence of local topography on the tide. This suggests that in deep water, vertical datums can be corrected using constant values. For this study, these corrections were applied as part of the vertical calibration (see *Vertical Calibration*), together with surveys that were not tide-corrected.

### Gridding

All point clouds were gridded using the inverse distance weight (IDW) algorithm with a search radius varying between 5 and 10 cells (depending on background noise and data density) and a distance exponent of 2. The IDW function was chosen over other interpolation algorithms because it is computationally efficient and ensures that the interpolated values remain within the data range. It should be noted that a distance exponent of 1 was used along the SE margins to account for the limited quality of the data. These values were selected to minimise the occurrence of gridding artefacts related to the anisotropy of the point distribution. Such anisotropy further limited the definition of the ideal cell size as it was not possible to use the average minimum distance between two points, divided by two, as recommended by Hengl^34^ for scattered point clouds. Indeed, the minimum distance between two points illustrates the point spacing along the survey lines but does not capture the spacing between these lines (Fig. 3). Instead, the cell size of each survey was set as the square root of the average area occupied by a point, defined as the total survey area divided by the number of datapoints. The total survey area corresponds to the envelope (polygon) of the survey point cloud, calculated using a maximum aggregation distance of 25 times the average minimum distance between two adjacent points. This approach generally produced seamless grids, but some of the navigation-derived bathymetric grids associated with the highest point distribution anisotropy still showed strong survey footprints. In such cases, and following a visual inspection of the output, a multiplier coefficient was applied to the cell size. The coefficient was gradually increased until the survey footprint became less apparent.

### Bathymetric calibration

#### Outlier filtering

**Fig. 6:**
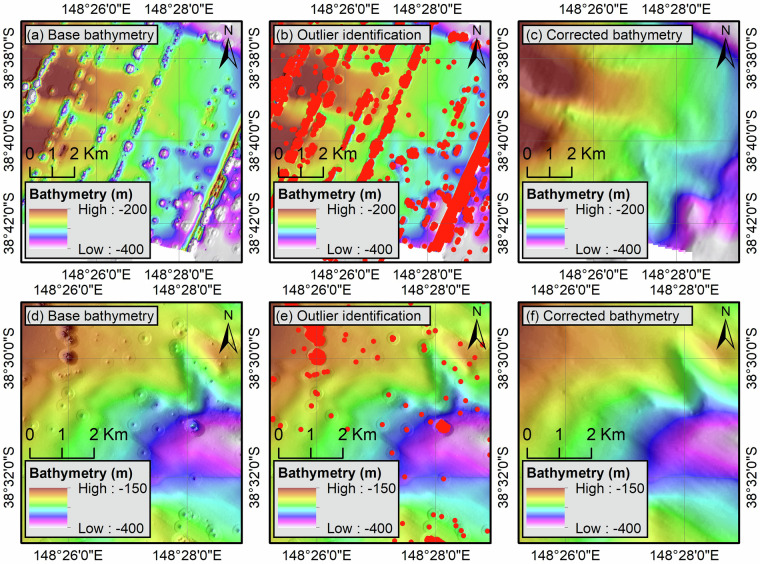
Illustration of the outlier removal process using the Angler (**a**–**c**) and South Marlin (**d–f**) 3D seismic surveys. The base bathymetry (**a,d**) is compared to source recordings to identify outliers based on threshold values (**b,e**) that are subsequently removed (**c,f**).

The bathymetric grids exhibit several artefacts in the form of either individual nuggets or trenches/ridges due to the presence of incorrect records in the point clouds (Fig. 6a,d). These points were identified by comparing the depth value of each depth measurement with the associated raster grid. If the difference amounts to more than 5% of the grid value, the depth measurement is considered an outlier and discarded (Fig. 6b–f).

In areas with steep topographic changes (e.g., along the continental slope), the threshold was set to 10% to limit the removal of false positives. This approach led to the identification of most erroneous records from navigation datasets but had only a marginal effect on reflection-derived bathymetry. Indeed, seismic traces are already corrected for outliers during geophysical processing and local artefacts, where present, are related to the use of low frequencies and sampling rates in shallow waters^2,35^ and extend over multiple acquisition lines, thus limiting the amount of discrepancy between depth measurements and the associated raster grids.

#### Cross calibration

**Fig. 7:**
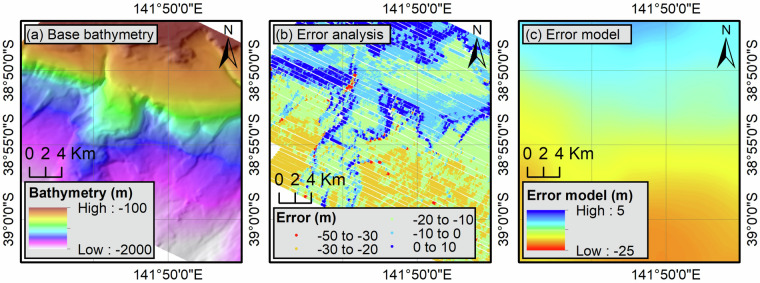
Illustration of the cross-calibration process using the OS02 seismic survey. Reflection-derived bathymetric grids (**a**) are compared with navigation depth soundings (**b**) to generate an error model (**c**) that is subsequently applied to the reflection-derived bathymetry.

Traditionally, 3D seismic surveys are acquired and processed to visualise hydrocarbon-bearing reservoir intervals typically located several thousand of metres below the seabed^35^. These data have a dominant frequency of an average of about 60 hertz^24^ and often exhibit a regional tilt or undulation of the seabed over several kilometres, illustrated by varying vertical offsets between adjacent surveys that can reach tens of metres. Navigation measurements, on the other hand, are recorded by the vessel’s echosounder and are therefore not affected by these regional artefacts. In order to maintain the spatial resolution of the reflection-derived bathymetry while benefiting from the vertical accuracy of the navigation data, the reflection-derived datasets were calibrated using navigation data following a method from Lebrec, *et al*.^2^ that was originally developed to calibrate satellite-derived bathymetry using hydrographic depth soundings. To do this, reflection-derived bathymetric depth values were extracted at the navigation depth sounding locations to calculate the absolute error between the two datasets (Fig. 7a,b). The values were then used to interpolate an error IDW grid using a bin size of 0.02 degrees and a distance exponent of 1 (Fig. 7c). These values were selected to obtain a smooth grid, only capturing regional trends rather than local discrepancies between navigation and reflection datasets. The resulting grid was then resampled to the resolution of the reflection-derived bathymetric grid using a bilinear interpolation, before being added to it to obtain corrected reflection-derived bathymetric grids. It should be noted that this step could only be conducted on surveys where available navigation data fully overlap the reflection data. As a result, significant offsets, particularly in the Bonaparte basin, may still be present between adjacent surveys. These were reduced as much as practical through vertical calibration (see next section) but this was not always possible.

#### Vertical calibration

Several surveys could not be reduced to a common vertical datum due to a lack of tidal corrections or source datum information in the metadata. Similarly, while the remaining surveys were reduced to EGM2008 based on available information, it is common to observe vertical offsets between adjacent surveys of a few metres (and rarely of up to several tens of metres). To reduce such uncertainties and to support seamless integration of the various surveys, all datasets were corrected using AHO depth soundings that had been converted from LAT to EGM2008 as reference points. The density and accuracy of AHO depth soundings decrease dramatically in areas away from urban areas or beyond the continental shelf edge and about a third of the surveys are intersected by fewer than five datapoints, hence limiting the representativeness of any error assessment. In such cases, AHO depth soundings were supplemented with depth values from overlapping MBES surveys and, where no other calibration points were available, with depth values from adjacent 3D surveys that had been previously calibrated. The definition of the calibration values was completed in an iterative process to ensure minimal discontinuities between adjacent surveys.

### Generation of regional compilations

To facilitate the use of the data, all bathymetric grids were merged to produce seamless seismic-derived bathymetric grids over four geographic areas around Australia comprising the: (1) southeast margins (i.e., Bass Strait, Gippsland Basin and Otway Basin); (2) southwest margins (i.e. Great Australian Bight Basin and Perth Basin); (3) Rowley Shelf; and (4) Browse Basin and Bonaparte Basins (Fig. 1). In most areas, multiple grids associated with varying resolutions and noise levels overlap, hence raising the question of which surveys to include, and under what conditions, to ensure that the most reliable grids are put forward in the compilations.

**Fig. 8:**
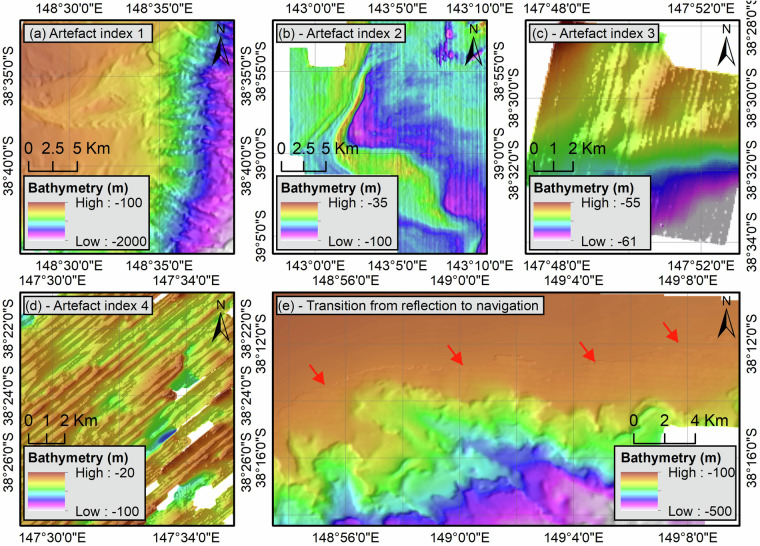
All bathymetric grids have been assigned an Artefact Index ranging from 1 to 4 (**a**–**d**) to reflect the quality of the data and facilitate their compilation. Reflection-derived seismic bathymetric grids sometimes show an improvement of the Artefact Index with increasing water depth. In such cases, shallow-water areas have been stitched with navigation-derived grids (**e**).

All bathymetric grids were sorted according to three criteria. First, all grids were visually inspected and assigned an artefact score ranging from 1 to 4 depending on the number of artefacts with: (1) no visible data artefacts and well-defined seabed morphologies (Fig. 8a); (2) a few visible data artefacts and not too distorted seabed morphologies (Fig. 8b); (3) strong data artefacts and distorted seabed morphologies (Fig. 8c); and (4) strong data artefacts and unrecognisable seabed morphologies (Fig. 8d). Second, within each category, the grids were ranked according to their cell size so that grids with the smaller cell size appeared above the others. Third, navigation-derived grids were prioritised over reflection-derived bathymetry in water depths less than 150 m given that the accuracy of reflection-derived bathymetry decreases in shallow water^2,15,35^. Additionally, when bathymetric grids had similar scores, the grid with the largest extent was preferred. In some cases, the quality of a grid can vary laterally due to data artefacts confined to a specific area. In particular, reflection-derived grids often have an artefact score of 1 in deep water which changes to 3 or even 4 in shallow water. In such instances, the grids were split into sub-grids and evaluated independently (Fig. 8e).

Lastly, before being merged, all bathymetric grids were resampled to 0.0003 × 0.0003 degrees (approximately 30 × 30 m at the equator) using bilinear interpolation. Considering that some navigation-derived bathymetric grids have a source resolution up to 10 times this value, resampling can lead to the formation of step-like artefacts highlighting the former grid resolution. To work around this, the bathymetric grids were resampled iteratively, dividing the cell size by two at each iteration, until the target cell size of 0.0003 degrees was achieved.

## Data Records

All datasets can be accessed via the AusSeabed Data Portal. The data asset includes four 0.0003 × 0.0003 degree (30 × 30 m at the equator) compilations as well as associated metadata and lineage file. The data can be accessed separately over the Bonaparte and Browse Basins 10.26186/147396^36^, the Southeast Margins 10.26186/148609^37^, the Southwest Margins 10.26186/148610^38^ and the Rowley Shelf 10.26186/148611^39^.

## Technical Validation

### Vertical accuracy

**Fig. 9:**
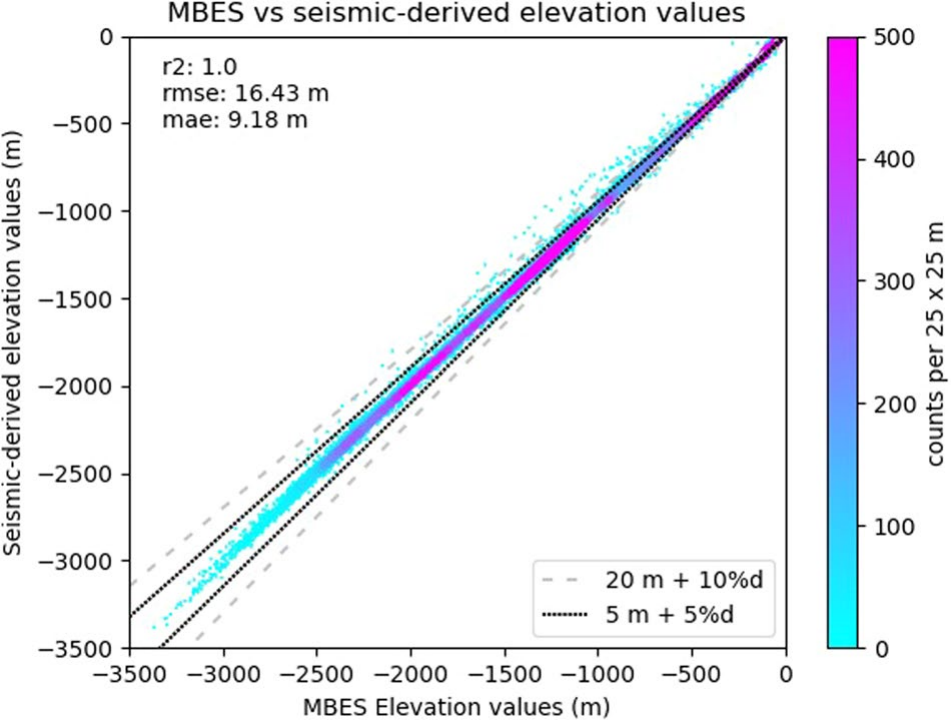
Comparison of MBES and seismic-derived bathymetry using 51,162 points. The black dotted line and grey dashed line represent IHO Total Vertical Uncertainty orders^40^.

Evaluating the vertical accuracy of individual bathymetric grids is a non-trivial task due to the lack of comparative datasets. Indeed, AHO depth soundings are used as part of the calibration process and therefore cannot be used as a reference. In this context, publicly available multibeam echosounder surveys are the only source of reliable depth soundings for comparison with seismic-derived bathymetric grids. Unfortunately, multibeam echosounder surveys are scarce, especially in shallow water, and overlap with few 3D seismic surveys precluding any analysis on a survey-by-survey basis. Furthermore, it is quite common to observe vertical offsets of up to tens of metres between adjacent multibeam echosounder surveys. For example, the average difference between multibeam echosounder surveys and AHO depth points along the Browse and Bonaparte Basins is 10.92 m indicating that multibeam echosounder surveys themselves have variable vertical accuracy. Considering these limitations, an estimate of the accuracy of the seismic-derived bathymetry was performed using the regional compilations and multibeam echosounder surveys, based on depth values extracted from both datasets along a 0.01-degree grid of points (approximately 1,000 m at the equator) for a total of 51,162 data points. The result indicates that seismic-derived bathymetric compilations have a mean average error of 9.18 m, with 96.6% and 99.5% of the points meeting the hydrographic accuracy of 5 m + 5%d (order 1^40^) and 20 m + 10%d (order 2^40^), respectively (Fig. 9). These results are similar to what was obtained by Power and Clarke^16^.

This value is subject to wide variability and should be contrasted with the amount of artefact, cell size, and in particular, navigation versus reflection sources. Navigation depth soundings are recorded by a dedicated echo sounder and, as such, represent the most reliable measurements. However, the anisotropy of the recordings (Fig. 3) means that large corridors remain largely unsurveyed and the grid cell sizes must be increased significantly to limit the generation of banded gridding artefacts, and may therefore not fully capture seabed conditions. Additionally, several navigation recordings did not include information about the sound velocity and tide corrections that were applied (or not applied) further increasing uncertainties. On the other hand, reflection-derived grids are associated with smaller cell sizes, in the range of 12.5 to 25 m which capture seabed features much more precisely. However, the relative height of seabed morphologies tends to be distorted with shallower depth, especially below 150 m, locally multiplying the relative heights by a factor of 5. A similar pattern has previously been observed in the Gulf of Mexico^15^ suggesting that these artefacts are ubiquitous in 3D seismic surveys, in line with previous descriptions^2,35^. A possible explanation could be that in shallow water, the seabed is poorly recorded due to the minimum receiver offsets and far field ranges of typical 3D seismic surveys, and is further masked by direct reflections and refractions

### Position accuracy

Modern 3D seismic acquisition vessels are equipped with differential global positioning systems which are associated with a positional accuracy of a few centimetres^24^. Older systems relied on reference stations with a lower accuracy, in the order of 1 to 3 metres^41^. In any case, such values remain below the minimum distance between two adjacent points and are therefore unlikely to affect the results in any significant way.

### On the nature of the first return

**Fig. 10:**
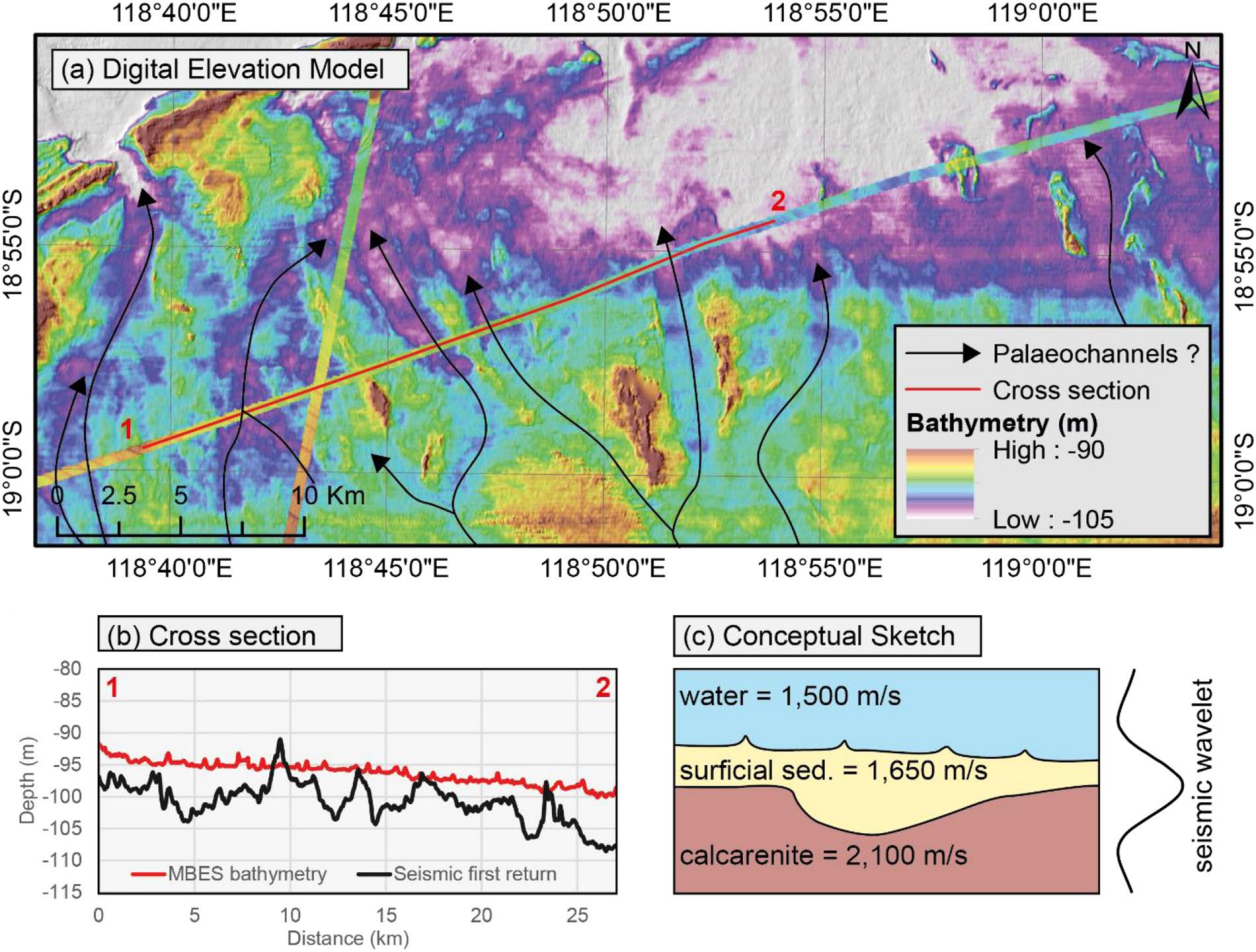
Illustration of the first seismic return. The bathymetry derived from the first return presents a number of palaeochannels that are not visible from the MBES bathymetry in both map view (**a**) and cross-section (**b**). Velocities from the publicly available Dorado-3 well completion report indicate that the velocity contrast (and hence acoustic impedance) between the surficial sediment and the underlying calcarenite is much higher than between the water and the surficial sediment. As a result, where the thickness of the surficial sediment is less than the seismic wavelength, the first return captures the calcarenite and not the seabed (**c**). Data courtesy of TGS.

Lastly, some uncertainties remain about the nature of the first return from seismic reflection. It is commonly assumed to be the seabed but the comparison of reflection-derived bathymetric grids with MBES data from the North West Shelf of Australia suggests that in some cases the first return may not correspond to the seafloor but to the top of the first lithified layer. Indeed, along the continental shelf, seabed features such as palaeochannels can be observed from the first return data but are completely absent from the MBES bathymetry (Fig. 10a,b).

Such a pattern could result from the evolution of layer densities and associated velocities with depth. Considering that the water has a velocity of about 1,500 m/s while the surficial sediments and the lithified layer have velocities of about 1,650 m/s and 2,100 m/s respectively, the transition from the surficial sediment to the lithified layer will result in a much higher contrast than the transition from the water to the surficial sediment (Fig. 10c). As a result, if the thickness of the surficial sediment is less than the length of the seismic pulse, the first return will capture the lithified layer instead of the seabed. This configuration is much more likely to be encountered in shallow water where successive eustatic cycles result in greater sediment variability than in deep water, further increasing the uncertainties in the shallow water reflection-derived bathymetry. This also means that, for a given area where such a pattern has been observed, if both MBES and reflection-derived bathymetry are available it may be possible to derive a thickness map of the surficial sediment.

## Usage Notes

**Fig. 11:**
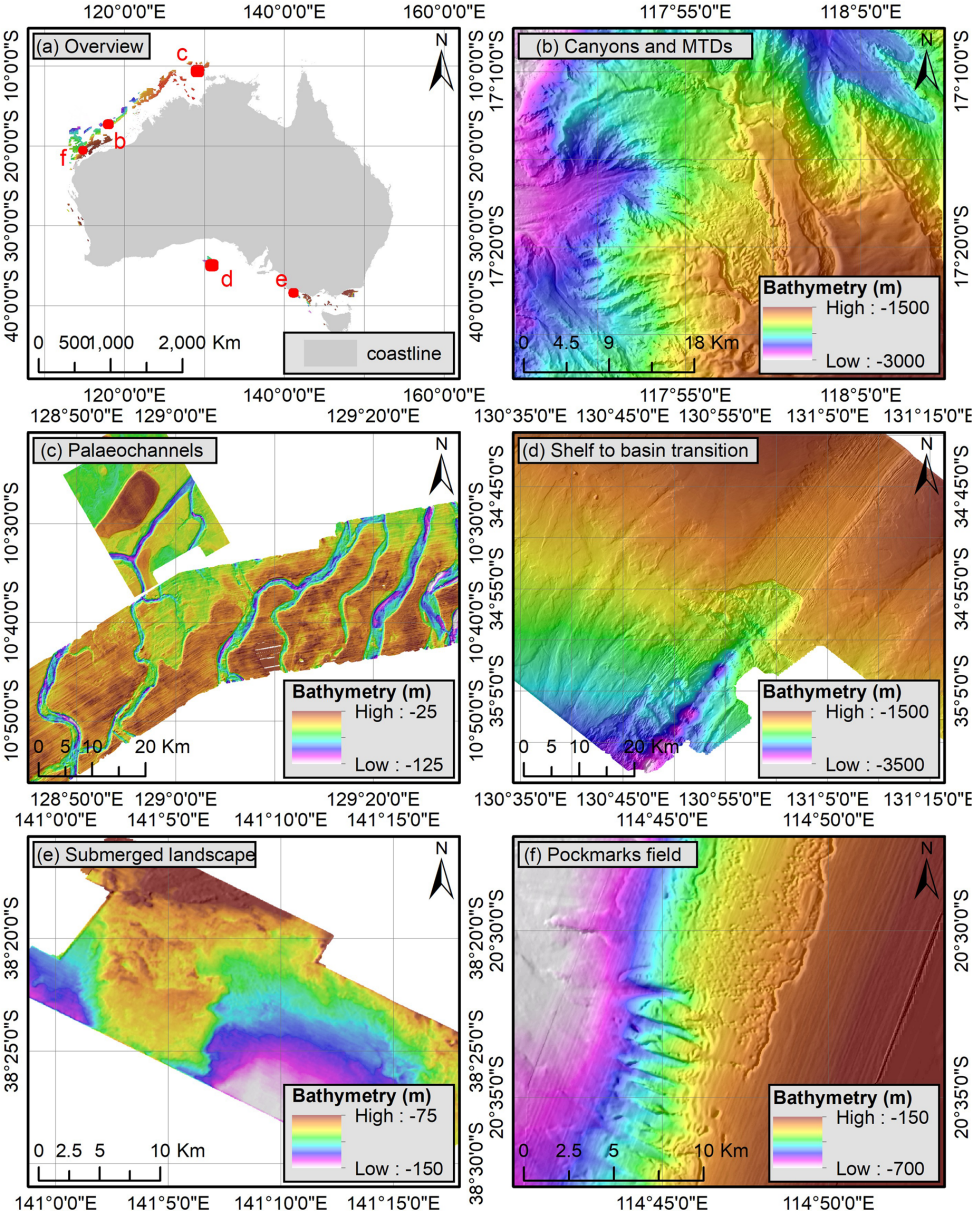
Examples of bathymetric features revealed by the seismic-derived bathymetric data asset. (**a**) Overview, (**b**) Canyons and mass transport deposits from the North West Shelf, (**c**) Relict tidal channels, (**d**) Gravity-driven features associated with the shelf to basin transition, (**e**) Ancient landscape from the Bass Strait, (**f**) Pockmark fields associated with extensive canyon systems and mass transport deposits.

The Australian margins host some of the world’s richest biodiversity hotspots, associated with key ecological features^42,43^, but remain largely unexplored^44^, as demonstrated by the recent discovery of a 500 m high reef along the Great Barrier Reef^45^ and modern ooid shoals in a deltaic environment along the North West Shelf^46^. In many cases, the data assets presented here cover areas that have never been surveyed before, hence enabling a broad range of oceanographic and seabed geomorphological studies (Fig. 11). In particular, the data cover large areas of the continental shelves of the Bass Strait and the North West Shelf which are expected to host submerged archaeological sites^21,47^ and exhibit evidence of past sea levels^18,48,49^. Similarly, the imaging of numerous canyons could contribute to a better understanding of geohazards and source-to-sink sediment transport across carbonate-dominated margins^50^. Furthermore, the integration of such datasets will be critical for the development of renewable energy which, due to their extent, cannot rely solely on site-specific data^4^. This is particularly important in carbonate-dominated environments where soil conditions are considered problematic^51,52^. In this regard, Lebrec, *et al*.^53^ demonstrated that the integration of regional bathymetry with climatic data can be used to predict the geotechnical properties of selected seabed features. Additionally, the analysis of the nature of the 3D seismic first return shows that, on continental shelves, it can be used to obtain thickness maps of the surficial sediments, which are an important input not only for offshore engineering studies^37^ but also to better understand marine habitats.

## Data Availability

The workflow presented in this manuscript was conducted using the Python programming language. Scripts were developed using publicly available libraries for performing raster and shapefile computations including Pandas, GeoPandas, and Numpy as well as the commercial library Arcpy that comes with Esri ArcGIS software. All processing steps can be performed manually using any GIS software. Scripts are available on GitHub: https://github.com/ausseabed/seismic-bathymetry. The Python scripts were supplemented by the AusCoastVDT^33^ and Doris freeware, respectively, to convert the data tidal datums and generate synthetic velocity profiles. Lastly, the commercial software Palaeoscan^TM^ was used to extract the first pick from seismic traces. Further details on the use, parameters and limitations of the software are presented in relevant subsections.
